# Effects of daily 24-gram doses of rice or whey protein on resistance training adaptations in trained males

**DOI:** 10.1186/s12970-020-00394-1

**Published:** 2020-12-01

**Authors:** Jessica M. Moon, Kayla M. Ratliff, Julia C. Blumkaitis, Patrick S. Harty, Hannah A. Zabriskie, Richard A. Stecker, Brad S. Currier, Andrew R. Jagim, Ralf Jäger, Martin Purpura, Chad M. Kerksick

**Affiliations:** 1 Exercise and Performance Nutrition Laboratory, School of Health Sciences, Lindenwood University, 209 S. Kingshighway, St. Charles, MO 63301 USA; 2 Energy Balance and Body Composition Laboratory, Department of Kinesiology & Sport Management, Texas Tech University, Lubbock, TX USA; 3 Department of Kinesiology, Towson University, Towson, MD USA; 4 Sports Medicine, Mayo Clinic Health System, Onalaska, WI USA; 5 Increnovo LLC, Milwaukee, WI USA

**Keywords:** Protein source, Supplementation, Rice, Whey, Plant proteins, Protein isolates, Fat-free mass, Body composition, Strength, Endurance, Performance, Efficacy

## Abstract

**Background:**

Large (48-g), isonitrogenous doses of rice and whey protein have previously been shown to stimulate similar adaptations to resistance training, but the impact of consuming smaller doses has yet to be compared. We evaluated the ability of 24-g doses of rice or whey protein concentrate to augment adaptations following 8 weeks of resistance training.

**Methods:**

Healthy resistance-trained males (*n* = 24, 32.8 ± 6.7 years, 179.3 ± 8.5 cm, 87.4 ± 8.5 kg, 27.2 ± 1.9 kg/m^2^, 27.8 ± 6.0% fat) were randomly assigned and matched according to fat-free mass to consume 24-g doses of rice (*n* = 12, Growing Naturals, LLC) or whey (n = 12, NutraBio Labs, Inc.) protein concentrate for 8 weeks while completing a standardized resistance training program. Body composition (DXA), muscular strength (one-repetition maximum [1RM]) and endurance (repetitions to fatigue [RTF] at 80% 1RM) using bench press (BP) and leg press (LP) exercises along with anaerobic capacity (Wingate) were assessed before and after the intervention. Subjects were asked to maintain regular dietary habits and record dietary intake every 2 weeks. Outcomes were assessed using 2 × 2 mixed (group x time) factorial ANOVA with repeated measures on time and independent samples t-tests using the change scores from baseline. A *p*-value of 0.05 and 95% confidence intervals on the changes between groups were used to determine outcomes.

**Results:**

No baseline differences (*p* > 0.05) were found for key body composition and performance outcomes. No changes (*p* > 0.05) in dietary status occurred within or between groups (34 ± 4 kcal/kg/day, 3.7 ± 0.77 g/kg/day, 1.31 ± 0.28 g/kg/day, 1.87 ± 0.23 g/kg/day) throughout the study for daily relative energy (34 ± 4 kcals/kg/day), carbohydrate (3.7 ± 0.77 g/kg/day), fat (1.31 ± 0.28 g/kg/day), and protein (1.87 ± 0.23 g/kg/day) intake. Significant main effects for time were revealed for body mass (*p* = 0.02), total body water (*p* = 0.01), lean mass (*p* = 0.008), fat-free mass (*p* = 0.007), BP 1RM (*p* = 0.02), BP volume (*p* = 0.04), and LP 1RM (*p* = 0.01). Changes between groups were similar for body mass (− 0.88, 2.03 kg, *p* = 0.42), fat-free mass (− 0.68, 1.99 kg, *p* = 0.32), lean mass (− 0.73, 1.91 kg, *p* = 0.37), fat mass (− 0.48, 1.02 kg, *p* = 0.46), and % fat (− 0.63, 0.71%, *p* = 0.90). No significant between group differences were seen for BP 1RM (− 13.8, 7.1 kg, *p* = 0.51), LP 1RM (− 38.8, 49.6 kg, *p* = 0.80), BP RTF (− 2.02, 0.35 reps, *p* = 0.16), LP RTF (− 1.7, 3.3 reps, *p* = 0.50), and Wingate peak power (− 72.5, 53.4 watts, *p* = 0.76) following the eight-week supplementation period.

**Conclusions:**

Eight weeks of daily isonitrogenous 24-g doses of rice or whey protein in combination with an eight-week resistance training program led to similar changes in body composition and performance outcomes. Retroactively registered on as NCT04411173.

## Background

Recommended protein intake levels to meet the requirements of nearly all healthy adults are set at 0.8 g of protein per kilogram of body mass per day [1]. However, professional organizations such as the American College of Sports Medicine (ACSM) and International Society of Sports Nutrition (ISSN) recommend that exercising adults should consume higher amounts of daily protein [2, 3]. In response to exercise and in the absence of feeding, rates of muscle protein synthesis (MPS) and muscle protein breakdown both increase with the overall net muscle protein balance remaining negative [4]. However, the acute consumption of an efficacious dose (i.e., 0.25–0.35 g/kg/dose) of high-quality protein has been consistently shown to stimulate rates of MPS leading to a positive net muscle protein balance [4–6]. The establishment of a positive net muscle protein balance through exercise and high-quality protein ingestion supports many exercise and nutrition strategies employed by individuals who wish to improve their body composition and/or increase their fat-free mass [5, 6].

Several protein sources derived from either animal (e.g., whey, casein, egg, beef, fish) or plant (e.g., soy, rice, pea, hemp) origins are available for individuals to consume and meet their daily protein needs. Assessments of protein quality routinely rate various protein sources based upon the amount and distribution of the essential amino acids, which are required for consumption in the diet and are known to operate as prerequisites for maximal stimulation of MPS [7, 8]. Protein sources can be further evaluated based upon their digestibility, bioavailability, rate of amino acid appearance, and relative amount of any given individual amino acid. Proteins are also characterized by more general health-related factors such as the presence of allergens, cholesterol, or saturated fat [3, 6]. Due to differing amino acid profiles, many different types of animal proteins, particularly milk proteins (whey and casein) rank high on these rating scales, while various plant proteins that have lower amounts of one or more of the essential amino acids routinely rank lower [9]. Specifically, leucine content has become commonly accepted as an additional means by which the quality of a protein can be assessed, since previous work has indicated leucine may exert an independent influence over the promotion of a positive nitrogen balance and stimulation of MPS [10, 11]. In particular, leucine in doses ranging from 1.7–3.5 g may be needed to optimally promote MPS [3, 6]. Therefore, the leucine content of varying protein sources has become a relevant consideration when choosing food-derived protein options that can go on to influence exercise training adaptations over time.

Previous work by Joy and colleagues [12] compared the effect of isonitrogenous doses of rice protein or whey protein on adaptations to resistance training, suggesting that both protein sources equally stimulated improvements in strength, performance, and fat-free mass [12]. While intriguing, these results lack ecological validity due to the large protein dose provided (48 g), which is substantially larger than typical ‘per serving’ doses ingested by consumers or those recommended in the scientific literature [3, 5, 6, 13, 14], notwithstanding recent work in nighttime protein feedings [15, 16]. Other research by Purpura et al. [17] compared the rate and magnitude of amino acid appearance after ingesting a single 48-g dose of a rice or whey protein isolate. Whey protein isolate stimulated a faster peak concentration of the essential amino acids, nonessential amino acids, and total amino acids. When total area under the curve was computed over a four-hour measurement window, whey isolate was responsible for a 6.8% greater level of amino acids when compared to changes seen with rice protein isolate ingestion, a difference that was not statistically significant. Moreover, when the time to peak concentrations were identified between the two sources of protein, whey protein isolate resulted in faster appearance of most amino acids with the exception of leucine, whereas rice protein ingestion stimulated a faster time to peak leucine concentration in comparison to whey [17].

In considering these outcomes, additional research needs to be completed to identify the potential efficacy of ingesting smaller, more ecologically valid doses of rice protein while performing a heavy resistance training program. These findings will extend the previous results of Joy et al. [12] and also provide potential implications of the previous work of Purpura et al. [17]. Therefore, the purpose of this study was to compare the effect of isocaloric and isonitrogenous (24-g) doses of rice or whey protein concentrate (~ 80% protein) on resistance training adaptations in young, healthy, resistance-trained men. It is hypothesized that there will be no differences in strength and body composition adaptations observed between study participants who supplement with rice or whey protein throughout the study protocol.

## Methods

### Experimental design

**Fig. 1:**
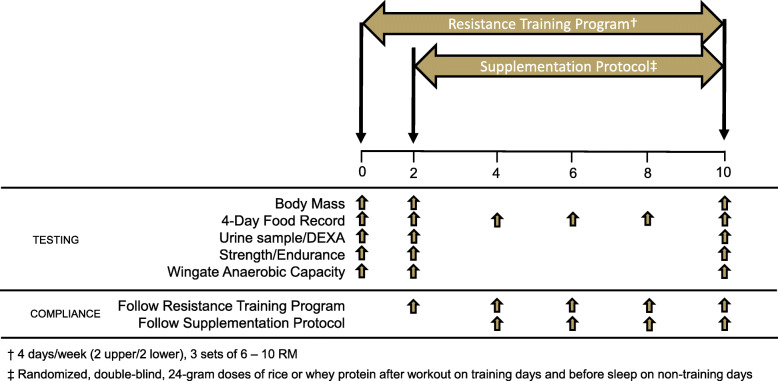
Research Design Overview

The study was conducted using a randomized, double-blind approach where participants were matched into groups according to fat-free mass. Participants were provided 24-g doses of rice or whey protein concentrate for an eight-week supplementation period that coincided with completion of a linear periodized, split-body resistance training program consisting of two upper body and two lower body workouts each week. On workout days, one dose of supplemental protein was ingested within 60 min of workout completion. On non-workout days, one dose was ingested within 60 min of going to bed. A total of 10 weeks of resistance training were completed over the entire study protocol. The first 2 weeks of resistance training (eight workouts) occurred before supplementation began and were completed to acclimate participants to the program and to initiate early neurological adaptations commonly seen with starting a new resistance training program. After completion of this run-in period, participants then began the supplementation protocol and continued to follow the resistance training program for an additional 8 weeks. Consequently, a total of 40 workouts were assigned in this protocol. Before and after the resistance training and supplementation period, body mass, body water (total, extracellular, intracellular), and body composition (fat mass, fat-free mass, lean mass, and % fat) were assessed. Changes in muscular strength (one-repetition maximum [1RM]) and muscular endurance (repetitions to fatigue at a load of 80% 1RM, [RTF]) were assessed using the bench press and leg press exercise, and Wingate anaerobic capacity tests were completed to assess anaerobic power. All anthropometric, body composition, and performance assessments were completed after 0, 2, and 10 of weeks of resistance training (Fig. 1). No supplementation occurred during the first 8 weeks of this protocol; consequently, all statistical analysis was completed on data collected starting at the beginning and end of supplementation. Participants were provided nutritional recommendations in order to ensure adequate energy (> 30 kcal/kg body mass/day) and protein consumption (> 1.5 g/kg/day) to facilitate positive training adaptations and reduce the potential influence of differing dietary intakes [3, 18]. Compliance cut-offs for removal for noncompliance were established at 90% compliance. This study was retroactively registered on clinicaltrials.gov as NCT04411173.

### Study participants

Healthy, resistance-trained males (*n* = 24, age: 32.8 ± 6.7 years, height: 179.3 ± 8.5 cm, weight: 87.4 ± 8.5 kg, body mass index: 27.2 ± 1.9 kg/m^2^, body fat %, via DXA: 21.7 ± 3.9% fat) participated in the study. To be eligible, participants reported at least 1 year of (self-reported) resistance training experience, could bench press > 1.0x their body weight, could leg press > 1.5x their body weight, and had a BMI less than 30 kg/m^2^. If a participant’s BMI was greater than 30 kg/m^2^, they were accepted in the study if their body fat percentage (determined by DXA) was less than 30% body fat. Additionally, participants were required to stop taking all nutritional supplements except for multi-vitamins for 30 days before participating in the study and for the entire duration of the protocol.

#### Anthropometric & Resting Assessments

Prior to all laboratory visits, participants fasted for at least 8 h and abstained from exercise, caffeine, nicotine, and alcohol for at least 24 h. Upon arrival during the initial assessment, participant height was assessed to the nearest ±0.5 cm using an analog wall-mounted stadiometer (HR-200, Tanita Corp, Inc., Tokyo, Japan) with their shoes removed and standing erect on flat feet. Body mass was measured prior to all study visits using a self-calibrating digital balance (Tanita BWB-627A, Tokyo, Japan) and was recorded to the nearest ±0.1 kg. Additionally, body masses recorded after 0 and 2 weeks were compared to ensure the participants were weight stable. Any participant whose body mass deviated by more than 2% during this time was excluded from participation. Heart rate as well as systolic and diastolic blood pressure were assessed using an automatic blood pressure monitor (OMRON BP785, Omron Corporation, Kyoto, Japan). All hemodynamic measurements were completed in a supine position after the study participant had arrived in the laboratory and rested quietly on an exam table for approximately 10 min. Participants were assessed for hydration status by providing a mid-flow urine sample analyzed by a handheld urine refractometer.

#### Body composition

##### Dual-energy X-ray absorptiometry

Body composition was assessed using dual-energy x-ray absorptiometry (DXA) (Hologic QDR Discovery A, Hologic, Inc., Bedford, MA, USA). A trained research assistant positioned all participants and manually analyzed all scans. Each day, the DXA was calibrated according to manufacturer recommendations and each scan was analyzed using the provided software (Hologic APEX Software, Version 4.5.3, Hologic Inc., Bedford, MA, USA) with the National Health and Nutrition Examination Survey (NHANES) analysis approach employed. Fat-free mass, lean mass, fat mass, and body fat percentage were recorded. Test-retest reliability has been previously established for DXA fat mass (CV: 1.26%, ICC: 0.99, SEM: 127.8 g) and DXA fat-free mass (CV: 0.75%, ICC: 0.99, SEM: 110.9 g) in cohort (*n* = 40) of healthy college-aged men and women.

##### Total body water

Total body water (TBW) was assessed using bioelectrical impedance spectroscopy (SFB7, Impedimed Corp., Carlsbad, CA). Each participant’s socks were removed to clean the dorsal surface of the foot and wrist prior to electrode placements. Two electrodes were placed on both the hand and foot. The proximal electrode for the foot was placed between the medial and lateral malleolus bones while the distal electrode was placed with the midpoint of the electrodes five cm apart. For the hand, researchers placed the proximal electrode on the midline of the ulnar styloid process with the distal electrode placed with the midpoints of the two electrodes spaced five cm apart. Once the electrodes were in place, researchers applied the four leads to their designated landmarks per manufacturer guidelines and researchers made certain the participants were not touching their sides and their legs were properly spaced apart. Three consecutive measurements were completed, averaged, and used for subsequent data analysis. TBW estimates produced by this unit have previously been validated against deuterium oxide dilution techniques in resistance-trained males [19]. Test-retest reliability has been previously established for bioelectrical impedance spectroscopy fat mass (CV: 5.86%, ICC: 0.98, SEM: 280.9 g) and fat-free mass (CV: 1.72%, ICC: 0.99, SEM: 285.1 g) in a cohort (*n* = 40) of healthy college-aged men and women.

#### Performance assessments

##### Maximal strength

To assess changes in muscular strength throughout the study protocol, each participant’s one-repetition maximum (1RM) was determined using the leg press and bench press exercises. Prior to 1RM determination, a standardized warm-up of simple stretches and whole-body movements spanning 5–10 min was completed. Using a protocol consistent with National Strength and Conditioning Association (NSCA) recommendations, participants completed one set of ten repetitions at 50% of their estimated 1RM. The warm-up continued in a progressive fashion (six repetitions at 70% of their perceived 1RM, four repetitions at 85% 1RM, and one repetition at 95% 1RM). Two minutes of rest were observed between each set. One-repetition sets were then completed with progressively increasing loads until a 1RM was determined. The 1RM for each exercise was determined within three to five one-repetition attempts and 2 min of rest were observed between each attempt. Post-testing 1RM assessment was completed using their previously established 1RM as a determinant for establishing loads throughout testing. Participants rested for 5 min between determination of their 1RM and completion of the next test. All leg press repetitions were completed using a commercial, 45-degree hip sled/leg press machine. For all leg press activities, foot position and hip angle was standardized by recording the heel position throughout testing and using these parameters for future 1RM determinations. Participants were required to keep their hands free of their knees, thighs, and torso and lower the weight until the knee angle reached approximately 90 degrees of flexion. All bench press repetitions were completed using a standard adjustable bench press and knurled bar (Rogue Fitness, Columbus, OH). Hand spacing was standardized for each attempt by recording the width of the hands for each repetition. In accordance with technique standards, participants were required to maintain five points of contact during all bench press repetitions and lower the bar to the sternum and press back until both elbows reached full extension. Two experienced research team members were present to ensure appropriate technique was maintained for both exercises throughout the testing.

##### Muscular endurance

Approximately 5 min after each respective 1RM determination, study participants completed an upper and lower body muscular endurance assessment. These assessments were completed using a load that corresponded to 80% of their 1RM for both the leg press and bench press exercises. Participants were instructed to complete one set with as many repetitions as they could until failure and were required to maintain appropriate lifting technique throughout all repetitions while being supervised by a research team member. Full range of motion was required for all repetitions. The test was stopped and completed repetitions were counted if participants paused for greater than 2 s between repetitions or technique failure occurred throughout any repetition. Total training volume (sets x repetitions performed with 80% 1RM × 80% 1RM load) for each exercise was computed and statistically analyzed. Further, data was normalized to one’s body mass and entered for statistical analysis as both the raw and normalized data. Participants rested for 5 min between completion of their repetitions to failure and completion of the next test. All performance assessments were completed in the exercise lab and supervised by trained research assistants.

##### Anaerobic capacity

After completion of muscular strength and endurance protocols and 5 min of rest, anaerobic capacity was assessed using the Wingate anaerobic capacity test on a Monark cycle ergometer (Ergomedic 894E, Vansboro, Sweden). The testing protocol began with a two-minute warm-up consisting of light pedaling (< 50 rpm) against zero resistance. The resistance for all Wingate testing was set at 7.5% of Week 0 body weight (kg) for each participant and was not changed for any subsequent test. Seat height and position was assessed during the initial test and was standardized for each subsequent use. After the warm-up, participants were provided a three-second count down and instructed to increase pedal speed and reach maximal speed. Trained investigators manually dropped the resistance when the participant reached their maximal speed, and the participant continued to pedal as fast as possible against their allotted resistance for the 30-s test. Upon completion of the test, the resistance was removed, and participants transitioned into a two-minute cool down on the bike against zero resistance. Peak power, average power and time to fatigue was computed and used as indicators of anaerobic power and fatigue resistance.

#### Resistance training program

A template of the resistance-training program is outlined in Table 1. Participants were provided with online training cards via email following completion of the Week 0 performance assessment. Participants updated their training log online each week to provide accountability for each workout. The program was designed as a linear, split-body periodization program with two upper-body and two lower-body workouts each week [20]. A progressive overload scheme was followed to facilitate increases in strength and muscle mass. For the first 6 weeks (weeks 1–6), each workout consisted of three sets of ten repetitions at a 10 RM load. On the final set of each exercise, participants performed as many repetitions as they were able. Following the autoregulatory model introduced by Mann et al. [21], if participants were able to complete 12 or more repetitions on their final set, they were instructed to increase the load for their next workout. During the final 4 weeks (weeks 7–10), each workout consisted of four sets of six repetitions to momentary muscle failure. Again, participants completed as many repetitions as they were able on their final set. If participants completed seven or more repetitions on their final set, they were assigned to the next highest load for their next workout [21]. One minute of rest in between sets was allotted for weeks 1–6, while 2 min of rest between each set were followed for weeks 7–10. Each resistance training session took approximately 60 min to complete. Completion of the program was not directly supervised. To maximize ecological validity, participants completed their workouts in the facility of their choosing, provided they had access to all equipment necessary to complete the exercises within the program. All study participants completed the first 2 weeks of the strength training protocol without supplementation for two primary reasons. First, to allow participants to identify what loads were needed to reach momentary muscular failure for each exercise on their third prescribed set. Second, this two-week period allowed for neuromuscular acclimation to the training stimulus, which may have occurred due to prescribing participants to follow a training program different than what they had been following.

**Table 1:** Sample resistance training program

Weeks	Day 1, Day 3	Day 2, Day 4
1–6^a^	Bench press, 3 × 10 RM Chest flies, 3 × 10 RM Lat pull, 3 × 10 RM Seated row, 3 × 10 RM Shoulder press, 3 × 10 RM Shoulder shrugs, 3 × 10 RM Biceps curls, 3 × 10 RM Triceps extensions, 3 × 10 RM	Back squat or leg press, 3 × 10 RM Leg extensions, 3 × 10 RM Romanian Deadlift, 3 × 10 RM Split Lunges, 3 × 10 RM Leg curls, 3 × 10 RM Calf raises, 3 × 10 RM Ab crunches, 3 × 25
7–10^b^	Bench press, 4 × 6 RM Chest flies, 4 × 6 RM Lat pull, 4 × 6 RM Seated row, 4 × 6 RM Shoulder press, 4 × 6 RM Shoulder shrugs, 4 × 6 RM Biceps curls, 4 × 6 RM Triceps extensions, 4 × 6 RM	Back squat or leg press, 4 × 6 RM Leg extensions, 4 × 6 RM Deadlift, 4 × 6 RM Lunges, 4 × 6 RM Leg curls, 4 × 6 RM Calf raises, 4 × 6 RM Ab crunches, 3 × 25

^a^One-minute rest between sets

^b^Two minutes rest between sets

To monitor compliance to the resistance-training program, participants were instructed to complete the online training cards (Google Sheets) weekly by selecting the exercise completed and filling out the number of reps and load used. Completion of logs was monitored weekly by research team members and emails were sent or phone calls made to facilitate compliance. In addition, participants were required to submit daily photographs of them in their gym either before or after their workouts. On each training day, participants would take a photograph of themselves at the gym and submit it through text, email, or an Instagram direct message. All training cards were reviewed, and photos were logged by laboratory staff weekly to monitor compliance to the training protocol. Compliance was calculated as the percentage of completed workouts. Participants were required to achieve and maintain at least 90% workout completion throughout the study. Compliance to recording their workout information and submitting pictures was determined to be 99.8% for the whey protein group and 99.4% for the rice protein group.

#### Dietary protocol

After the Week 0 performance assessment, participants were provided daily dietary recommendations. A range of daily caloric needs was estimated for each study participant by calculating resting energy expenditure using an average of the Harris-Benedict [22] and Mifflin-St. Joer [23] formulas and then multiplying that value by an activity factor of 1.6 and 1.8. Participants were also instructed to maintain a daily protein intake of 1.6 to 1.8 g of protein per kilogram of body mass [3]. Participants were required to log their dietary intake at least 4 days each week through MyFitnessPal over the course of the study. Dietary intake data can be found in Table 2.

**Table 2:** Dietary Variables

Variable	Group	Week 2	Week 4	Week 6	Week 8	Week 10	Within Group (*p*)	G x T (*p*)
Caloric Intake (kcal/day)	Rice	2583 ± 930	2678 ± 941	2895 ± 436	2609 ± 921	2361 ± 1178	0.57	0.34
	Whey	3168 ± 336	2786 ± 921	2483 ± 1211	2655 ± 891	2672 ± 919	0.34	
Relative Caloric Intake (kcal/kg)	Rice	29.1 ± 10.3	–	–	–	26.2 ± 12.7	0.57	0.61
	Whey	36.7 ± 4.9	–	–	–	30.7 ± 10.6	0.08	
Carbohydrate (g/day)	Rice	280 ± 107	302 ± 119	323 ± 80	294 ± 125	262 ± 130	0.61	0.36
	Whey	332 ± 98	311 ± 139	267 ± 158	278 ± 134	287 ± 139	0.41	
Relative Carbohydrate (g/kg/day)	Rice	3.16 ± 1.19	–	–	–	2.91 ± 1.43	0.67	0.61
	Whey	3.85 ± 1.17	–	–	–	3.27 ± 1.48	0.11	
Protein (g/day)	Rice	157 ± 62	138 ± 50	157 ± 29	135.8 ± 53	130 ± 67	0.47	0.57
	Whey	167 ± 27	153 ± 57	141 ± 70	147 ± 50	153 ± 50	0.63	
Relative Protein (g/kg)	Rice	1.79 ± 0.76	–	–	–	1.43 ± 0.70	0.25	0.57
	Whey	1.93 ± 0.26	–	–	–	1.76 ± 0.60	0.33	
Fat (g/day)	Rice	95 ± 37	101 ± 40	109 ± 28	97 ± 47	86 ± 46	0.60	0.16
	Whey	129 ± 32	104 ± 40	93 ± 49	106 ± 39	104 ± 36	0.18	
Relative Fat (g/kg)	Rice	1.07 ± 0.40	–	–	–	0.95 ± 0.50	0.58	0.47
	Whey	1.50 ± 0.38	–	–	–	1.20 ± 0.44	0.07	

All data presented as Mean ± SD; *p* = probability of making a type I error

All variables relative to body mass use body mass obtained during Week 2

To achieve compliance to the dietary recommendations outlined above, study participants used MyFitnessPal (Under Armour, Baltimore, MD) to set up profiles and track their dietary intake. In addition, study participants were provided a binder that outlined their recommended energy and protein intakes throughout the study protocol. Participants were given examples of how to successfully fill out their food information in addition to graphic-based examples of portion size estimators. Laboratory staff reviewed the food records weekly and documented if the participants met both caloric and protein requirements 4 days per week. Finally, when participants visited the laboratory every 2 weeks to receive more supplements, their records were reviewed, and questions were asked regarding compliance to the diet, exercise, and supplementation.

#### Supplementation protocol

Following performance re-assessment 2 weeks after beginning the resistance-training program, participants were randomly assigned to the whey protein or rice protein groups in a double-blind fashion. Participants were then block randomized within each protein group based on their week two fat-free mass using an online randomization software program (Random Allocation Software). Participants were provided with 15 doses of 24 g of rice protein (33.6 g of chocolate flavored rice protein concentrate, Growing Naturals, LLC, Axiom Foods) or 24 g of whey protein (32.6 g of chocolate flavored whey protein concentrate, NutraBio Labs, Inc.). Each 24-g dose of protein was mixed with 6–12 fluid ounces of cold water. On workout days, participants ingested one dose within 60 min of completing their workout. On non-workout days, one dose was ingested within 60 min of going to bed. The total protein content and amino acid profile for both protein supplements were analyzed by an independent laboratory, (Eurofins Food Chemistry Testing US, Inc., Madison, WI, USA) and can be found in Table 3. Both protein groups were isocaloric and isonitrogenous. Additionally, samples of the rice protein and whey protein were third-party analyzed by LGC Sciences, Inc. (Lexington, KY, www.Igcgroup.com) for the presence of banned substances. Each supplement was analyzed for over 214 compounds of interest using a variety of techniques including GC-MS/MS, LC-MS/MS, and LC-HRMS. All tests were found to be negative for all compounds targeted within LGC’s core supplement screen.

**Table 3:** Amino Acid Composition

	Whey Protein		Rice Protein	
	AA (mg/serving)	AA (mg/g protein)	AA (mg/serving)	AA (mg/g protein)
Aspartic acid	2512	104.2	2213	89.2
Threonine	1625	67.4	909	36.7
Serine	1193	49.5	1272	51.3
Glutamic Acid	3966	164.6	4319	174.2
Proline	1424	59.1	1181	47.6
Glycine	423	17.6	1114	44.9
Alanine	1210	50.2	1446	58.3
Valine	1327	55.1	1466	59.1
Isoleucine	1520	63.1	1114	44.9
Leucine	2509	104.1	2118	85.4
Tyrosine	771	32.0	1374	55.4
Phenylalanine	717	29.8	1378	55.6
Lysine	2247	93.2	705	28.4
Histidine	411	17.1	553	22.3
Arginine	673	28.0	2006	80.9
Cystine	585	24.3	581	23.4
Methionine	533	22.1	681	27.5
Tryptophan	455	18.9	352	14.2
**Essential AAs (mg)**	11,344	471	9276	374
**Total BCAAs (mg)**	5356	222	4698	189
**Total Amino Acids (g)**	24.1	1	24.8	1

To monitor compliance to the supplementation regimen, participants were required to visit the laboratory every 2 weeks where they were given an additional 15 sachets of their assigned protein. This time was taken to review compliance to the dietary recommendations, completion of dietary records, training program, and adverse events. In addition, on training and non-training days, participants were required to submit a photograph of themselves with the supplement in a clear container prior to its consumption. All photos were submitted via text, email, or Instagram direct message, in an identical fashion to the evidentiary photographs for the resistance training program. Supplement photos were logged by laboratory staff to ensure that participants were regularly consuming their protein doses. For the next three visits (Weeks 4, 6, 8), participants were required to return to the lab to exchange their empty sachets for 15 new doses. To achieve compliance, participants were required to submit a returned sachet and submit a photograph. Compliance was calculated as the percentage of days in which compliance was achieved divided by the total number of days in the protocol. Compliance to the supplementation protocol was calculated to be 99.4% for the whey protein group and 99.6% for the rice protein group.

### Statistical analysis

All analyses were completed using Microsoft Excel and the Statistical Package for the Social Sciences (v23; SPSS Inc., Chicago IL). A priori statistical analysis using G-Power revealed that achieving an effect size of 0.25 with an alpha level of 0.05 and power of 0.80 would require a total sample size of 22 participants. Before any statistical tests are performed, data was analyzed for normality. All non-normal data were log-transformed prior to analysis. For all statistical tests, data were considered statistically significant when the probability of a type I error was 0.05 or less. Primary endpoints for this investigation were fat-free mass and leg press 1RM. Secondary endpoints were fat mass, lean mass, and % body fat along with bench press 1RM, bench press repetitions to fatigue, leg press repetitions to fatigue, leg press volume, peak anaerobic power, mean anaerobic power, and rate of fatigue. 2 × 2 mixed factorial (group x time) ANOVAs with repeated measures on time were used to determine any statistically significant differences for time and group main effects and group x time interaction effects for all primary and secondary outcomes. 2 × 5 repeated measures ANOVA were used for all group x time, time, and group effects for all dietary variables. In instances, where baseline group differences were evident, ANCOVA analysis was completed with the respective baseline value as a covariate. All data are presented as means ± standard deviations.

## Results

### Baseline differences

**Fig. 2:**
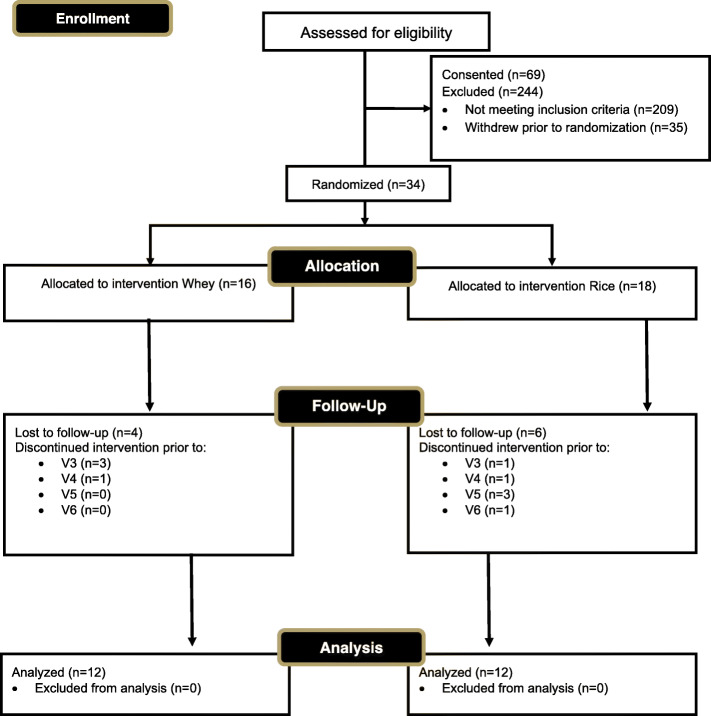
CONSORT diagram

In response to recruitment flyers, study team members made contact with 313 individuals. A large majority (*n* = 244) were excluded, while 69 people were consented. Of the exclusions, 209 did not meet inclusion criteria while an additional 35 withdrew prior to randomization. The remaining 34 participants were randomized, with 16 being assigned to ingest whey protein and 18 being assigned to ingest rice protein. Four people in the whey group were lost to follow up due to failure to maintain contact or non-compliance, while six people in the rice group were lost to follow-up due to failure to maintain contact with research team member or non-compliance with the protocol. This led to two groups of 12 participants in each group that were included in the analysis (Fig. 2). Prior to starting the resistance training and supplementation protocols, independent t-tests revealed no baseline differences (*p* > 0.05) between groups for age (95% CI: − 3.8, 7.6 years, *p* = 0.50), height (95% CI: − 7.7, 7.1 cm, *p* = 0.93), body mass (95% CI: − 9.1, 5.5 kg, *p* = 0.61), body mass index (− 2.13, 1.20 kg/m^2^, *p* = 0.57), systolic blood pressure (95% CI: − 10.0, 1.8 mmHg, *p* = 0.16), diastolic blood pressure (95% CI: − 1.8, 7.0 mmHg, *p* = 0.23), resting heart rate (95% CI: − 7.2, 5.4 beats/min, *p* = 0.77), % body fat (95% CI: − 4.81, 1.78% fat, *p* = 0.35), leg press 1RM (95% CI: − 57, 34 kg, *p* = 0.61), and bench press 1RM (95% CI: − 9.3, 19.5 kg, *p* = 0.47).

### Dietary intake

Dietary data was collected at two-week intervals throughout the study protocol and was analyzed using both normalized (per week 2 body mass in kg) and non-normalized data. Since body mass was only collected at the beginning and end of supplementation, normalized data was analyzed using 2 × 2 mixed factorial ANOVA with repeated measures of time. All non-normalized data was subsequently analyzed using 2 × 5 mixed factorial ANOVA with repeated measures on time. As seen in Table 2, group x time interactions, time, and group effects for all non-normalized data for energy (group x time: *p* = 0.34; group: *p* = 0.54; time: *p* = 0.67), carbohydrate (group x time: *p* = 0.36; group: *p* = 0.94; time: *p* = 0.73), protein (group x time: *p* = 0.57; group: *p* = 0.51; time: *p* = 0.52), and fat (group x time: *p* = 0.16; group: *p* = 0.36; time: *p* = 0.55) were non-significant. Additionally, similar outcomes were revealed when all data was represented relative to each person’s recorded body mass: normalized energy (group x time: *p* = 0.61; group: *p* = 0.94; time: *p* = 0.15), normalized carbohydrate (group x time: *p* = 0.61; group: *p* = 0.24; time: *p* = 0.21), normalized protein (group x time: *p* = 0.57; group: *p* = 0.21; time: *p* = 0.14), and normalized fat (group x time: *p* = 0.47; group: *p* = 0.01; time: *p* = 0.11) (Table 2).

Baseline differences between groups were identified that revealed greater amounts of normalized energy (mean difference: 7.6 ± 3.3 kcal/kg, 95% CI: 0.72, 14.4 kcal/kg, *p* = 0.03) and normalized fat intake (mean difference: 0.43 ± 0.16 g/kg, 95% CI: 0.10, 0.76 g/kg, *p* = 0.01) for the whey protein group when compared to the rice protein group. Follow-up ANCOVA were completed on this data with each respective baseline values as a covariate to assess changes in normalized energy and fat intakes between groups. ANCOVA results indicated no between-group differences for the normalized energy (*p* = 0.37) and fat intake (*p* = 0.28).

### Anthropometrics and body water

Table 4 highlights main effects for group x time interaction, time, and group effects for body mass, total body water, and body composition variables. Changes in body mass indicated a non-significant group x time interaction (*p* = 0.42; Mean Difference: 0.58 ± 0.70 kg; 95% CI: − 0.88, 2.03 kg, *d* = − 0.07), a significant main effect for time (*p* = 0.02), and a non-significant group effect (*p* = 0.59). As anticipated, similar changes were identified for body mass index within and between groups (data not shown, see Table 4). Changes in total body water indicated a non-significant group x time interaction (*p* = 0.51; Mean Difference: − 0.52 ± 0.79 l; 95% CI: − 2.16, 1.11 l, *d* = 0.12), significant main effect for time (*p* = 0.01), and a non-significant group effect (*p* = 0.61) (See Table 4).

**Table 4:** Hemodynamic and Body Composition Variables

						Group x Time	Time	Group
Variable	Group	Week 2	Week 10	ES	95% CI	(p)	(*p*)	(*p*)
Heart Rate (beats/min)	Whey Rice	63.7 ± 8.1 58.0 ± 9.6	63.8 ± 9.0 59.0 ± 10.2	0.10	(−7.2, 5.4)	0.77	0.73	0.15
Systolic BP (mm Hg)	Whey Rice	124.9 ± 10.7 125.4 ± 5.9	120.5 ± 10.3 125.1 ± 6.3	0.48	(−10.0, 1.8)	0.16	0.11	0.44
Diastolic BP (mm Hg)	Whey Rice	69.1 ± 8.6 68.1 ± 8.7	69.8 ± 9.0 66.2 ± 8.8	0.29	(− 1.8, 7.0)	0.23	0.56	0.51
Body Mass (kg)	Whey Rice	87.0 ± 8.7 89.2 ± 8.1	88.2 ± 9.1† 89.7 ± 7.8	− 0.07	(− 0.88, 2.03)	0.42	0.02	0.59
Body Mass Index (kg/m^2^)	Whey Rice	27.2 ± 1.9 27.8 ± 1.9	27.5 ± 1.9† 27.9 ± 1.8	− 0.08	(− 0.34, 0.65)	0.51	0.07	0.51
Total Body Water (L)	Whey Rice	49.7 ± 4.5 50.4 ± 4.2	50.5 ± 4.1† 51.7 ± 4.4†	0.12	(−2.16, 1.11)	0.51	0.01	0.61
DXA Bone Content (kg)	Whey Rice	3.05 ± 0.40 3.13 ± 0.30	3.08 ± 0.38 3.10 ± 0.28	0.20	(0.01, 0.12)	0.02	0.91	0.70
DXA Lean Mass (kg)	Whey Rice	63.4 ± 5.3 64.7 ± 6.3	64.5 ± 5.7† 65.3 ± 6.2†	− 0.10	(− 0.73, 1.91)	0.37	0.01	0.60
DXA Fat Mass (kg)	Whey Rice	17.3 ± 4.9 18.4 ± 3.7	17.5 ± 4.7 18.4 ± 3.4	− 0.06	(− 0.48, 1.02)	0.46	0.60	0.54
DXA Fat-Free Mass (kg)	Whey Rice	65.9 ± 5.6 67.2 ± 6.6	67.0 ± 5.8† 67.8 ± 6.5†	−0.11	(− 0.68, 1.99)	0.32	0.01	0.60
DXA Body Fat (%)	Whey Rice	20.6 ± 4.2 21.5 ± 3.8	20.5 ± 3.9 21.4 ± 3.8	0.01	(− 0.63, 0.71)	0.90	0.49	0.58
Dry Lean Mass (kg)	Whey Rice	16.7 ± 2.1 17.6 ± 3.9	17.1 ± 2.5 16.9 ± 3.7	− 0.35	(− 0.46, 2.69)	0.16	0.72	0.78

† = Significant change (*p* < 0.05) within each group from each group’s respective baseline; *Time* Main effect for time; *p* probability level of making Type I error; *95% CI* 95% confidence intervals were computed on the observed changes from baseline between groups; *ES* Effect size calculated as ([Week 10 Rice Protein – Week 2 Rice Protein] – [Week 10 Whey Protein – Week 2 Whey Protein]) / Pooled SD. *DXA* Dual-energy x-ray absorptiometry

### Body composition

**Fig. 3:**
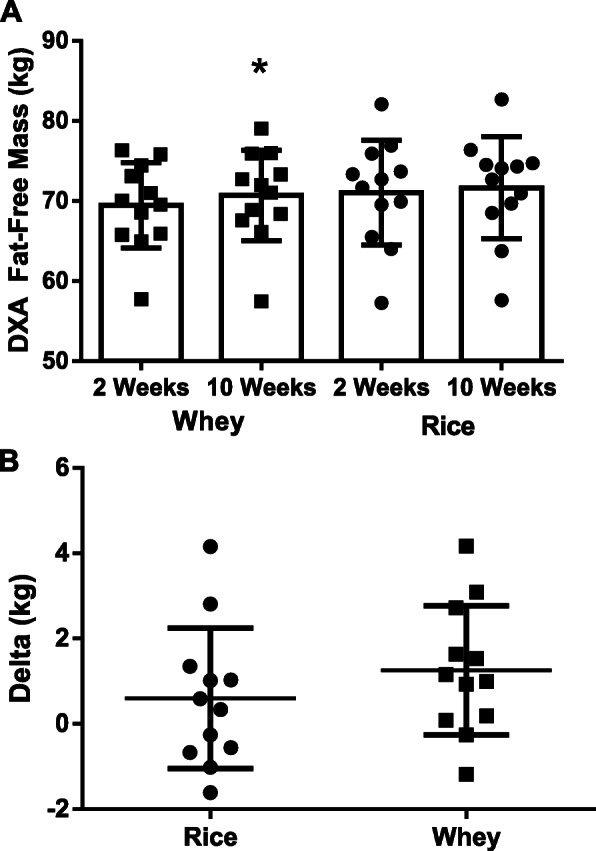
(Sub-Panel **a** & **b**): DXA fat-free mass (in kilograms) in rice and whey protein supplemented groups. Panel **a**: Raw data (Rice = 0.05 ± 4.8% change; Whey = 1.5 ± 4.5% change); Panel **b**: Individual response data. All data is presented as means ± SD. * = Different from within-group week 0 value

Changes in DXA lean mass indicated a non-significant group x time interaction (*p* = 0.37; Mean Difference: 0.59 ± 0.64 kg; 95% CI: − 0.73, 1.91 kg, *d* = − 0.10), a significant main effect for time (*p* = 0.01), and non-significant group effect (*p* = 0.60). Changes in dry lean mass (DXA lean mass – total body water) indicated a non-significant group x time interaction (*p* = 0.16, Mean Difference; 1.11 ± 0.76, 95% CI: − 0.46, 2.69 kg, d = − 0.35), time (*p* = 0.72), and group effect (*p* = 0.16). Changes in DXA fat mass indicated no group x time interaction (*p* = 0.46; Mean Difference: 0.27 ± 0.36 kg; 95% CI: − 0.48, 1.02 kg, *d* = − 0.06), time (*p* = 0.60), or group effects (*p* = 0.54) for fat mass. Fat-free mass (Fig. 3a and b) changes identified no significant group x time interaction (*p* = 0.32; Mean Difference: 0.66 ± 0.64 kg; 95% CI: − 0.68, 1.99 kg, *d* = − 0.11) and time effect (*p* = 0.01) and no significant main effect for group (*p* = 0.60) was realized (Table 4). Changes in DXA percent fat changes indicated no group x time interaction (*p* = 0.90; Mean Difference: 0.04 ± 0.32% fat; 95% CI: − 0.63, 0.71% fat, *d* = 0.01), time (*p* = 0.49), or group (*p* = 0.58) effect. Bone mineral content changes did yield a significant group x time interaction (*p* = 0.02; Mean Difference: 0.07 ± 0.03 kg; 95% CI: 0.01, 0.12 kg, *d* = 0.20) while no significant main effect for time (*p* = 0.91) or group (*p* = 0.70) was found.

### Exercise performance

#### Muscular strength

**Fig. 4:**
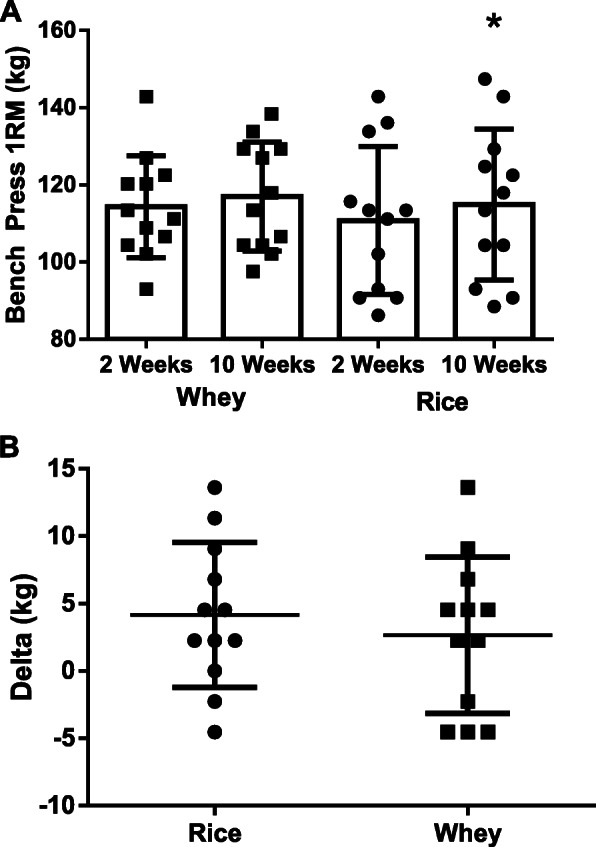
(Sub-Panel **a** & **b**): Bench-press one-repetition maximum (1RM) in rice and whey protein supplemented groups. Panel **a**: Raw data (Rice = 3.9 ± 4.9% change; Whey = 2.4 ± 5.0% change); Panel **b**: Individual response data. All data is presented as means ± SD. * = Different from within-group week 0 value

As seen in Table 5, changes in leg press 1RM indicated a non-significant group x time interaction (*p* = 0.80; Mean Difference: 5.42 ± 21.3 kg; 95% CI: − 38.7, 49.6 kg, *d* = 0.05), significant main effect for time (*p* < 0.001), and a non-significant group effect (*p* = 0.50). Within-group changes indicated both the rice and whey protein groups experienced significant improvements in their leg press 1RM at week 10 (*p* < 0.001). Changes in bench press 1RM (Fig. 4a and b) indicated a non-significant group x time interaction (*p* = 0.51; Mean Difference: − 3.33 ± 5.03 kg; 95% CI: − 13.8, 7.09 kg, *d* = 0.09), significant main effect for time (*p* < 0.001), and non-significant main effect for group (*p* = 0.68).

**Table 5:** Performance Variables

Variable	Group	Week 2	Week 10	ES	(95% CI)	*Group x Time* *(p)*	Time(*p*)	Group (*p*)
Bench Press 1RM (kg)	Whey Rice	114.4 ± 13.2 110.8 ± 19.2	117.0 ± 14.1 114.9 ± 19.5†	0.09	(−13.8, 7.1)	0.51	0.01	0.68
Bench Press Reps to Fatigue	Whey Rice	7.3 ± 1.4 6.8 ± 2.3	7.1 ± 1.7 7.4 ± 1.8	− 0.46	(− 2.02, 0.35)	0.16	0.57	0.91
Bench Press Volume	Whey Rice	1829 ± 307 1617 ± 487	1818 ± 449 1847 ± 444†	0.56	(− 556, 76)	0.13	0.16	0.56
Leg Press 1RM (kg)	Whey Rice	315 ± 42 331 ± 60	342 ± 41† 355 ± 62†	0.05	(−38.8, 49.6)	0.80	< 0.001	0.50
Leg Press Reps to Fatigue	Whey Rice	12.3 ± 3.1 10.6 ± 3.5	11.3 ± 4.4 8.8 ± 2.7	− 0.24	(−1.68, 3.35)	0.50	0.03	0.12
Leg Press Volume	Whey Rice	8538 ± 2487 7542 ± 2323	8438 ± 3276 6822 ± 2591	− 0.23	(− 1289, 2530)	0.51	0.38	0.20
Wingate Peak Power (W/kg)	Whey Rice	9.5 ± 1.0 9.7 ± 1.2	9.3 ± 1.0 9.7 ± 1.3	0.16	(− 0.91, 0.54)	0.60	0.54	0.53
Wingate Mean Power (W/kg)	Whey Rice	7.4 ± 0.7 7.5 ± 1.0	7.2 ± 0.7 7.3 ± 0.9	− 0.02	(− 0.34, 0.32)	0.94	0.12	0.77
Wingate Power Drop (watts)	Whey Rice	403 ± 102 433 ± 111	405 ± 107 433 ± 104	−0.02	(−98, 101)	0.97	0.96	0.44

† = Significant change (*p* < 0.05) within each group from each group’s respective baseline; *Time* Main effect for time; *p* probability level of making Type I error; 95% CI = 95% confidence intervals were computed on the observed changes from baseline between groups; ES = Effect size calculated as ([Week 10 Rice Protein – Week 2 Rice Protein] – [Week 10 Whey Protein – Week 2 Whey Protein]) / Pooled SD. Wingate power drop = Wingate maximum power – Wingate minimum power

#### Muscular endurance

Changes in leg press repetitions to fatigue indicated a non-significant group x time interaction (*p* = 0.50; Mean Difference: 0.83 ± 1.21 reps; 95% CI: − 1.68, 3.35 reps, *d* = − 0.24), significant main effect for time (*p* = 0.03), and non-significant main effect for group (*p* = 0.12). Changes in bench press repetitions to fatigue indicated a non-significant group x time interaction (*p* = 0.16; Mean Difference: − 0.83 ± 0.57 reps; 95% CI: − 2.02, 0.35 reps, *d* = − 0.46), non-significant main effect for time (*p* = 0.57), a non-significant main effect for group (*p* = 0.91). Bench press volume (80% 1RM x reps performed) was calculated at each study visit as a measure of muscular endurance. No group x time interaction (*p* = 0.13), time (*p* = 0.16), or group effects (*p* = 0.56) were identified. Leg press volume was computed in a similar fashion (1RM x reps performed) and no main effect for time (*p* = 0.38), group (*p* = 0.20), or group x time interaction was observed. (*p* = 0.51). In addition, total resistance training volume (sets x reps x load) was calculated for the entire eight-week protocol. No significant differences between group were found for upper-body (Whey: 195,806 ± 34,992 kg vs. Rice: 191,327 ± 41,215 kg, *p* = 0.78), lower-body (Whey: 229,736 ± 72,732 kg vs. Rice: 240,564 ± 54,455 kg, *p* = 0.69), and total volume (Whey: 425,542 ± 104,564 kg vs. Rice: 431,891 ± 91,615 kg, *p* = 0.88).

#### Anaerobic capacity

Peak power, mean power, and rate of fatigue were assessed in response to completion of the Wingate anaerobic capacity test. All power data was normalized to each person’s body mass in kg before being analyzed statistically. Changes in normalized peak power (watts/kg) indicated a non-significant group x time interaction (*p* = 0.76; Mean Difference: − 9.6 ± 30.4 watts/kg; 95% CI: − 72.5, 53.4 watts/kg), time (*p* = 0.54), or group (*p* = 0.53) effect. Changes in normalized mean power (watts/kg) indicated a non-significant group x time interaction (*p* = 0.94; Mean Difference: − 0.01 ± 0.16 watts/kg; 95% CI: − 0.34, 0.32 watts/kg), time (*p* = 0.12), and group (*p* = 0.77) effects. Changes in Wingate power drop (maximum power – minimum power) indicated a non-significant group x time interaction (*p* = 0.97; Mean Difference: 1.62 ± 48.0 watts; 95% CI: − 98.0, 101.3 watt), time (*p* = 0.96), or group (*p* = 0.44) effect.

## Discussion

We sought to examine if daily supplementation with 24-g doses of rice or whey protein concentrate during an eight-week resistance training program differentially impacts changes in strength and body composition in resistance-trained men. Primary outcomes of interest included changes in upper- and lower-body strength and DXA-determined fat-free mass, while secondary outcomes included changes in muscular endurance, resistance training volume, anaerobic power, fat mass, percent fat, bone mass, and body water. Findings relating to the primary outcomes indicated statistically significant improvements in body mass, total body water, lean mass, fat-free mass (Fig. 3), bench press 1RM (Fig. 4), and leg press 1RM over time with no differences between protein source. Moreover, no differences between protein sources were identified for all primary and secondary variables.

Previous research has established that rates of MPS and muscle protein breakdown increase in response to an acute bout of resistance training [4, 24]. Further, acute resistance training responses indicate that breakdown rates increase to a larger magnitude, resulting in a net negative muscle protein balance [24]. The provision of amino acids in the form of free amino acids or dietary proteins in dosages that provide 8–12 g of the essential amino acids promote maximal rates of MPS [25], ultimately resulting in a net positive muscle protein balance [25]. The quality of provided proteins can differ depending on the status of several factors, such as the amount of amino acids provided (especially the essential amino acids), the proportion at which those amino acids are delivered, and digestibility of the protein [5]. In general, plant-derived protein sources are considered to be of a lower quality as they typically are comprised of lower amounts of total essential amino acids, are low in one or more of the essential amino acids, and have lower levels of protein digestibility [9].

In accordance with the findings of Joy et al. [12], results from the present study identified significant improvements in body mass (*p* = 0.42; Mean Difference: 0.58 ± 0.70 kg; 95% CI: − 0.88, 2.03 kg, *d* = − 0.07) and fat-free mass (*p* = 0.32; Mean Difference: 0.66 ± 0.64 kg; 95% CI: − 0.68, 1.99 kg, *d* = − 0.11, Fig. 3) with no differences between protein sources. No significant group x time interactions were observed for any of the performance outcomes that were measured (Fig. 4, Table 5). Furthermore, findings from the present study align with the work of Babault et al. [26] who randomized 161 males between the ages of 18–35 years in a double-blind fashion to consume two daily 25-g doses (50 g total) of pea protein, whey protein, or placebo in conjunction a 12-week resistance-training program. The authors concluded that protein supplementation increased muscle thickness more than placebo, with these differences reaching statistical significance in the weakest individuals, but no differences being present between the protein groups. While our findings align with some of the recent work involving plant proteins and their ability to stimulate resistance training adaptations, our results contradict earlier scientific reports suggesting plant-based proteins, mainly soy protein, may not be suitable sources to potentiate resistance-training induced improvements in strength and body composition, and the majority [27–29], but not all [30, 31], of that work suggests whey protein is superior to soy protein. We also reported that whey protein was responsible for significantly greater increases in bone mineral content as measured by DXA (Table 4). A recent review by Deane et al. [32] highlighted several studies that demonstrate an increase in protein intake is commonly associated with improvements in bone status, but more research is needed regarding the impact of various protein sources before more definitive conclusions can be reached. Additionally, the length of our investigation would be considered by many to be too short to appropriately assess changes in bone status, so readers are encouraged to consider these factors prior to drawing firm conclusions surrounding the ability or inability of our two protein sources to impact bone status.

The primary basis for the reported inferiority of plant-based proteins focuses is their lower essential amino acid content and reduced digestibility. While the latter was not evaluated in the present study, a third-party independent analysis of each test protein revealed that when compared to rice protein, whey protein delivered approximately 26% more essential amino acids (471 vs. 374 mg of essential amino acids per gram of protein) and 22% more leucine (104.1 vs. 85.4 mg of leucine per gram of protein) (Table 3). Leucine is well accepted for its ability to stimulate the translation of cellular (including myofibrillar) proteins [33, 34], but the saturating dosage continues to be refined in humans. Using an animal model, Norton and colleagues [11, 35] suggested that a 2–3 g dose may be needed to stimulate protein translation, while previous work in healthy humans by Moore and colleagues [36] indicated a plateau of MPS rates beyond a 20-g dose of egg protein (which contains approximately two grams of leucine). Additionally, Churchward-Venne et al. [10] examined the acute and prolonged changes in MPS rates after ingesting a 25-g dose of whey protein or a 6.25-g dose of whey with added leucine (to make the provided leucine equivalent to a 25-g dose). These authors concluded that a lower dose of whey protein with added leucine similarly stimulated MPS, but that the 25-g dose was best suited to increase muscle protein accretion. In this respect, other sources of protein that have lower amounts of leucine and other amino acids may not be able to stimulate acute increases in MPS to the same degree, but might be equal when it comes to stimulating accretion of skeletal muscle protein. In the present study, a dose of 2.1 g of leucine was provided, while the leucine content delivered in the Babault study [26] is approximated (based on amino acid content data presented in their paper) to have delivered 1.6 g during each of the two daily doses provided in the study. While more research is needed, the combination of findings from these studies seem to indicate that supplemental doses of plant proteins (rice and pea specifically) can similarly promote resistance training adaptations when compared to supplemental doses of whey protein of identical energy and protein content. Researchers have posited that the leucine dose needed to maximally stimulate MPS may progressively lower with chronic resistance training and protein supplementation, provided adequate daily protein intake is consumed (1.6–1.75 g/kg body mass). Previously, Phillips and colleagues [37] reported on the changes that occur in protein metabolism in trained versus untrained individuals and revealed that greater turnover occurs in untrained participants. Thus, the requirements of leucine may change as training status progresses. Certainly, future work is needed to explore this possibility.

Readers of our paper are cautioned to closely consider some key limitations that exist within the present study. First, while consistent with other training studies of this nature [12, 38, 39], the duration of the supplementation and resistance training program in the present study was only 8 weeks. In this respect, it is acknowledged that a longer investigation may have allowed for a greater difference in the protein groups, which may have led to a statistically significant difference being identified between the groups. Second, the number of our subjects, while suitable to yield small between-group effects was still low for this study design. As a result, we may not have had enough power to appropriately identify between-group outcomes. However, our overall changes in fat-free mass accretion were similar in magnitude to those reported by Cermak et al. [40] in a meta-analysis of studies investigating the impact of protein supplementation on resistance training, but are smaller than other studies that were longer in duration [27, 41]. As such, readers are cautioned from drawing straight-line conclusions that rice and whey protein are equal until longer and larger investigations have been completed. Some may view our lack of direct exercise training supervision as a limitation, as previous work has indicated that strength increases are greater when direct supervision occurs [42]. However, the impact of supervision on body composition changes remains undetermined. Regardless, while participants in the present study were not directly supervised, they were required to submit daily photos after completing each workout and submit a separate photo of their daily supplementation consumption. In addition, participants were required to log their nutrition, and every 2 weeks returned their empty sachets and received a new supply of their assigned supplements. Further, these visits were used to counsel participants on meeting their assigned nutritional goals and reviewed the loads they were using as part of their exercise program. As seen in Table 2, participants were generally able to achieve appropriate levels of energy (29.3–30.8 kcal/kg body mass) and protein (1.6–1.75 g/kg body mass) to facilitate fat-free mass accretion. Unfortunately, we did not measure body mass at these two-week check points, which would have allowed for bi-weekly calculation of relative nutritional intake, but calculations using baseline body mass levels allow for suitable insight. Additionally, the raw (non-normalized) nutritional information can be found in Table 2.

A key strength to this investigation was the randomized, double-blind approach with an isocaloric and isonitrogenous protein control group. Additionally, to minimize the acute increases in strength commonly observed when someone begins a new exercise program, all participants completed eight workouts (four upper-body, four lower-body) prior to beginning their assigned supplement. As previously discussed, good compliance was accomplished or maintained for our prescribed nutritional (See Table 2) and resistance training regimens. Future research should explore longer investigations that incorporate more direct measures of body composition (muscle thickness and fiber cross-sectional area via skeletal muscle biopsy and ultrasound) and skeletal muscle protein turnover. In conclusion, the current investigation compared the impact of consuming 24-g doses of rice and whey protein concentrate while resistance training for 8 weeks. It was revealed that statistically similar changes in body composition changes and exercise performance were observed between the protein groups. While our results are limited by our study duration and sample size, initial evidence provided by this investigation suggests that supplementing with whey or rice protein concentrate may yield similar changes to body composition and performance over an eight-week period in healthy, resistance-trained men.

## Data Availability

Please contact corresponding author for additional data. Only blinded, de-identified data may be provided upon request with an appropriate and substantiated justification for any such request.
